# Real-World Safety and Tolerability of Low-Intensity Repetitive Transcranial Magnetic Stimulation in Fibromyalgia: A Multicenter Observational Cohort Study

**DOI:** 10.3390/jcm15124452

**Published:** 2026-06-09

**Authors:** Nazario Felix-Gonzalez, Jose-Maria Gomez-Arguelles, Ceferino Maestu-Unturbe

**Affiliations:** 1 Departamento de Arquitectura y Tecnología de Sistemas Informáticos, Escuela Tecnica Superior de Ingenieros Informaticos, Universidad Politecnica de Madrid, 28223 Madrid, Spain; 2 Unidad de Neurología, Hospital Universitario Quironsalud Madrid, 28223 Madrid, Spain; jmgarguelles@yahoo.es; 3 Centro de Tecnologia Biomedica, Universidad Politecnica de Madrid, 28223 Madrid, Spain; ceferino.maestu@ctb.upm.es

**Keywords:** fibromyalgia, neuromodulation, low-intensity rTMS, real-world evidence, safety, tolerability

## Abstract

**Background/Objectives**: Low-intensity repetitive transcranial magnetic stimulation (Li-rTMS) is an emerging non-invasive neuromodulation approach for fibromyalgia; however, large-scale data on safety and tolerability in routine clinical settings remain limited. This study aimed to evaluate the safety and tolerability of Li-rTMS in patients with fibromyalgia treated in outpatient practice. **Methods**: A retrospective multicentre observational cohort study was conducted using routinely collected clinical data from nine outpatient centres (November 2020–March 2026). Safety analyses included all patients initiating Li-rTMS treatment (intention-to-treat cohort, n = 1381). The protocol consisted of eight stimulation sessions delivered using a CE-certified medical device. Patient-reported tolerability and perceived symptom changes in three predefined domains (headache, generalized pain, and sleep) were assessed using a standardized 0–10 scale and analysed descriptively, with stratification by age group. **Results**: Li-rTMS was generally well tolerated across all age groups, including those >65 years. High-intensity symptom worsening was infrequent (headache 3.8%, generalized pain 6.1%, sleep disturbances 2.1%), predominantly mild and self-limited. No serious adverse events were spontaneously reported or clinically identified during routine clinical follow-up, within the limitations inherent to a real-world passive registry framework. Exploratory observations from patient-initiated recall sessions did not suggest an increased adverse symptom burden with repeated exposure. **Conclusions**: Li-rTMS demonstrated a favourable patient-reported tolerability profile among patients with available follow-up data, characterized by a low frequency of transient high-intensity symptom worsening and the absence of serious adverse events spontaneously reported or clinically identified during routine, non-systematic follow-up. These findings support the feasibility of repeated Li-rTMS administration in outpatient settings; however, due to the observational design and non-systematic follow-up, no conclusions regarding clinical efficacy can be drawn.

## 1. Introduction

Fibromyalgia (FM) is a chronic syndrome characterized by widespread musculoskeletal pain accompanied by fatigue, sleep disturbances, cognitive dysfunction, and emotional distress, leading to substantial impairment in quality of life and functional capacity [1,2,3]. The condition is considered part of the broader spectrum of central sensitization disorders, in which altered nociceptive processing and dysregulation of pain modulation pathways are believed to play key pathophysiological roles [4,5]. FM affects approximately 2–4% of the general population and occurs predominantly in women, representing a significant clinical and socioeconomic burden worldwide [1,6].

Current management strategies for FM remain challenging due to the heterogeneous and fluctuating nature of symptoms. Pharmacological approaches frequently provide limited or inconsistent benefits and are often associated with poor long-term adherence. Consequently, non-pharmacological and multimodal approaches, including exercise-based interventions, cognitive and behavioral therapies, and neuromodulation techniques, have gained increasing attention as complementary therapeutic strategies [3,7].

Among non-invasive neuromodulation approaches, repetitive transcranial magnetic stimulation (rTMS) has emerged as a promising option for modulating cortical excitability and pain-related neural networks in fibromyalgia (FM) [8,9,10,11]. However, available evidence remains heterogeneous, particularly for conventional high-intensity protocols, which have shown variable clinical outcomes and present practical limitations related to cost, accessibility, treatment complexity, and tolerability [9,10,11].

Low-intensity repetitive transcranial magnetic stimulation (Li-rTMS) has been proposed as an alternative neuromodulation approach using substantially lower magnetic field intensities than conventional rTMS [12,13]. Experimental and clinical studies have suggested that low-intensity magnetic stimulation may influence neuronal activity and cortical network dynamics without requiring suprathreshold stimulation [12,13]. Preliminary data in FM have reported potential improvements in pain-related and sleep-related outcomes, along with a favorable tolerability profile and feasibility for repeated administration [14,15,16].

Despite growing interest in Li-rTMS, important uncertainties remain regarding its safety and tolerability profile clinical use. Most available studies are based on small samples, controlled research settings, or limited follow-up periods [14,15,16]. Evidence on repeated exposure, patient-initiated maintenance sessions, and safety across different age groups is still limited.

This gap is especially relevant in chronic conditions such as fibromyalgia, where long-term adherence is essential and tolerability plays a major role in treatment feasibility, as poorly tolerated interventions are more likely to be discontinued.

Observational studies conducted in routine care settings can complement controlled trials by providing information on treatment tolerability in heterogeneous patient populations. In this context, the present multicenter observational cohort study was designed to evaluate the safety and tolerability of Li-rTMS in patients with FM. This study reports data from a large multicentre cohort of 1381 patients, providing extensive real-world information on Li-rTMS tolerability. Additionally, exploratory analyses were conducted to describe tolerability during patient-initiated recall sessions.

## 2. Materials and Methods

### 2.1. Study Design and Setting

A retrospective multicenter observational cohort study was conducted using routinely collected clinical data from nine outpatient centers specialized in chronic pain management between November 2020 and March 2026.

No experimental procedures or protocol-driven interventions were introduced beyond standard clinical care. The study was conducted and reported in accordance with the Strengthening the Reporting of Observational Studies in Epidemiology (STROBE) recommendations for observational cohort studies.

Given the observational design, the study was not based on formal hypothesis testing but aimed to descriptively evaluate the safety and tolerability profile of Li-rTMS in real-world clinical practice.

### 2.2. Participants and Eligibility Criteria

Eligible participants were adults aged 18 years or older with a clinical diagnosis of FM according to the 2010/2016 American College of Rheumatology (ACR) criteria [2]. Patients were included if they initiated the Li-rTMS treatment protocol during the study period and completed the initial pre-treatment assessment before stimulation.

Patients were excluded from treatment according to routine clinical safety criteria, including epilepsy or seizure disorders, severe uncontrolled psychiatric conditions, pregnancy or breastfeeding, implanted electronic medical devices, or other contraindications for magnetic stimulation established by the treating physician.

The initial database included 1446 clinical records. Data cleaning procedures were performed to ensure record integrity and remove duplicated or incomplete entries. The final intention-to-treat (ITT) cohort used for safety analyses included 1381 unique patients.

Patients attending at least one patient-initiated recall session following completion of the induction protocol were additionally identified for exploratory descriptive analyses related to repeated Li-rTMS exposure under routine clinical practice conditions.

### 2.3. Li-rTMS Intervention Protocol

Li-rTMS was delivered using a commercially available CE-certified Class IIa medical device (MINESTIM^®^, CTB, Madrid, Spain) intended for low-field transcranial magnetic stimulation in fibromyalgia-associated pain management. The device operates according to MDR Rule 9 and incorporates predefined manufacturer-programmed stimulation parameters.

From an engineering and verification standpoint, the electrical performance of the device was characterized through direct measurements conducted during functional testing by the technical responsible team at the manufacturer. These measurements confirmed that the stimulation system delivers a peak-to-peak current of approximately 800 µA following a square waveform at 8 Hz. The current flows through a network of 32 microcoils integrated into a helmet-based applicator, spatially distributed over the scalp according to a modified international 10–10 system, providing global and symmetrical cranial coverage. The coils are electrically connected in series configuration that ensures homogeneous current distribution under standardized operating conditions, with device tolerances maintained within ±10%.

Although direct measurement of magnetic flux density was not performed, the magnetic field generated by each microcoil was estimated from the measured current and coil geometry using standard electromagnetic formulations. Based on these calculations, the magnetic flux density at the coil level is in the order of approximately 1–2 µT (≈1.5 µT). These values represent theoretical engineering estimates under standardized operating conditions rather than direct in vivo biophysical measurements. This level is several orders of magnitude lower than the millitesla (mT) to tesla (T) range typically used in conventional high-intensity rTMS systems. Consequently, these values should be interpreted strictly as order-of-magnitude engineering estimates rather than direct measurements in biological tissues. The authors recognize that future physical investigations incorporating direct laboratory magnetic field measurements are required to conclusively validate these calculations and further strengthen the technical characterization of the device.

Participants underwent an induction protocol consisting of eight Li-rTMS sessions of 20 min each, administered under routine outpatient conditions. All sessions were supervised by trained healthcare personnel previously instructed in device operation and standardized treatment procedures. No anesthesia, sedation, or analgesic premedication was required.

The selection of stimulation frequency (8 Hz) and session duration (20 min) follows the manufacturer-defined protocol and is consistent with previous experimental and clinical studies on low-intensity magnetic stimulation. Frequencies in the low alpha range (8–10 Hz) have been associated with modulation of cortical network activity and functional brain rhythms without inducing direct neuronal depolarization. In the context of Li-rTMS, such frequencies are considered suitable for producing neuromodulatory effects while maintaining a favourable safety profile.

The session duration of 20 min was selected to ensure adequate exposure time for cumulative neuromodulatory effects under low-intensity conditions, while remaining well within established safety limits for repetitive magnetic stimulation. Given that Li-rTMS operates at substantially lower field strengths than conventional high-intensity rTMS, the applied frequency and duration are not associated with the safety constraints typically linked to suprathreshold stimulation protocols, such as seizure risk or motor threshold considerations.

Treatment sessions were conducted under controlled environmental conditions using non-ferromagnetic examination beds and low-electromagnetic-noise environments routinely employed for Li-rTMS administration. Standard sensory isolation measures, including ear protection and visual shielding, were applied according to the device operating protocol.

Following completion of the induction phase, additional recall sessions were available upon patient request as part of routine clinical management. Recall visits were not protocolized and were analyzed descriptively as exploratory observations.

### 2.4. Data Collection

Clinical data were collected within a post-market clinical follow-up (PMCF) program across specialized outpatient centres. All participating sites followed a standardized data collection workflow to ensure consistency in treatment delivery, assessment timing, and data recording.

Data were collected using a unified electronic questionnaire implemented on a secure digital platform (Google Forms). The questionnaire included predefined mandatory fields and was identical across all sites.

The questionnaire was administered by trained nursing personnel, who were specifically trained in both the Li-rTMS procedure and the PMCF data collection protocol. Data entry was performed using password-protected tablet devices with restricted access.

Patients completed the questionnaire prospectively during routine visits prior to selected treatment sessions. Responses were subsequently reviewed and validated by the treating physician before inclusion in the centralized PMCF database, ensuring data completeness and clinical oversight.

Assessments were conducted at predefined milestones: (1) baseline (prior to the first session), (2) intermediate (prior to the fourth session), and (3) end-of-treatment (prior to the eighth session). Additional data were collected during optional patient-initiated recall sessions.

The baseline assessment was used to characterize the study cohort but did not include treatment-related symptom evaluation, as no Li-rTMS exposure had occurred. Subsequent assessments captured patient-reported responses to treatment.

To enhance transparency and reproducibility, the PMCF workflow, questionnaire, and a representative anonymized dataset are publicly available in an open-access repository [17].

Data were stored in a centralized encrypted database hosted on institutional servers at the Universidad Politécnica de Madrid (UPM), with restricted access controlled by the project’s Technical Director. Data cleaning, preprocessing, and statistical analyses were performed using a standardized automated pipeline to ensure consistency and reproducibility.

### 2.5. Data Management

All clinical data were anonymized at the source prior to analysis using unique patient identification codes assigned by each participating center. No directly identifiable personal information was included in the analytical dataset.

Following physician validation, data were transferred to a centralized PMCF database for aggregation and analysis. The anonymized dataset was stored on a secure institutional cloud platform compliant with Regulation (EU) 2016/679 (General Data Protection Regulation, GDPR).

Data processing and statistical analyses were performed by the study investigators using the anonymized dataset, ensuring consistent handling of information and preventing access to patient identifiers throughout the analytical process.

This structured, multi-level data management approach—including trained staff involvement, physician validation, and centralized data handling—was designed to ensure data integrity, minimize variability across centers, and support the reliability of the study findings.

### 2.6. Safety and Tolerability Outcomes

Safety and tolerability outcomes were derived from structured patient-reported responses collected at predefined clinical assessment timepoints, rather than through a formal adverse event reporting system. Accordingly, outcomes reflect patient-perceived changes in symptoms under routine clinical conditions rather than formally adjudicated safety surveillance data.

Assessments focused on three predefined symptom domains: (1) headache, (2) generalized pain, and (3) sleep-related disturbances. These domains were evaluated using a standardized 0–10 numerical rating scale (NRS), designed to assess perceived change in symptoms relative to the pre-treatment condition rather than absolute symptom severity. In this scale, 0 indicates maximum improvement, 5 no change, and 10 maximum worsening. “Generalized pain” refers to overall perceived widespread pain typical of fibromyalgia, expressed as change from baseline.

Data were collected at predefined treatment milestones: baseline (prior to the first session), intermediate (prior to the fourth session), and end-of-treatment (prior to the eighth session), as well as during optional patient-initiated recall sessions. The baseline assessment was used to characterize the study cohort and did not include treatment-related evaluation. At subsequent visits, patients rated their symptom changes based on the preceding treatment sessions.

Each patient contributed one assessment per domain at each timepoint; therefore, outcomes are reported on a per-patient basis rather than per session. For descriptive analysis, responses were categorized as improvement (0–4), no change (5), or worsening (6–10).

Tolerability was operationally defined using transient high-intensity symptom worsening, defined as scores ≥8 in the headache or generalized pain domains at a given assessment. These responses were interpreted as clinically relevant short-term worsening occurring during or shortly after Li-rTMS exposure, based on routine clinical evaluation and physician validation.

Data were collected prospectively using a standardized electronic questionnaire administered by trained nursing personnel at predefined visits (Sessions 1, 4, and 8) prior to clinical evaluation. Patients did not complete the tolerability scale at baseline, as no stimulation had yet occurred. At intermediate and end-of-treatment visits, responses reflected the tolerability of the preceding sessions. Any major or unexpected clinical events not captured by the questionnaire were documented if spontaneously reported by patients during subsequent medical follow-up visits.

This approach captures patient-reported tolerability outcomes rather than formally adjudicated adverse events derived from active standardized surveillance systems. Accordingly, the absence of serious adverse events within this cohort should be interpreted within the context of routine, non-systematic clinical follow-up, where no clinically significant treatment-related complications were spontaneously reported or clinically identified.

Exploratory analyses were performed using data from patient-initiated recall sessions to describe symptom evolution and tolerability during repeated Li-rTMS exposure. As these assessments were non-protocolized and patient-driven, they were analysed descriptively and considered subject to potential selection bias.

### 2.7. Data Curation and Cleansing Protocol

Prior to statistical modeling, data extracted from the federated multicenter database underwent a multi-stage deterministic curation pipeline to ensure data integrity, structural consistency, and longitudinal traceability.

The curation process was systematically structured as follows:

- Data Aggregation and Standardization: Raw electronic data capture (EDC) sheets from all participating clinical centers were consolidated into a unified relational structure. Variables were normalized, and clinical site codes were cross-verified against institutional registry logs.
- Deduplication Audit: To avoid artificial statistical inflation and administrative noise, an automated deduplication protocol was applied. Records were flagged as redundant if they shared identical unique Clinical History Numbers (IP) alongside overlapping timestamps or duplicate questionnaire submissions within the same evaluation window. These multi-entry redundancies were systematically reviewed and collapsed into single, validated baseline records.
- Completeness and Quality Filters: A strict case-complete auditing protocol was enforced. Records missing critical baseline identifiers—such as biological sex, age, clinical centre origin, or core initial safety and symptomatic metrics—were excluded from subsequent analyses. This deterministic filtering approach was chosen to prevent the introduction of imputation biases in the initial demographic characterization.
- Longitudinal Traceability: Patients who successfully fulfilled the baseline data integrity criteria were assigned a verified, anonymized tracking identifier. This identifier was utilized to dynamically map individuals across all subsequent follow-up intervals—including intermediate assessments, acute treatment termination, and patient-initiated maintenance (recall) sessions—ensuring strict consistency throughout the study timeline.

### 2.8. Statistical Analysis

Descriptive statistics were used to summarize demographic characteristics and patient-reported outcomes. Continuous variables are presented as mean ± standard deviation (SD), and categorical variables as frequencies and percentages.

Analyses were performed using all available data at each assessment timepoint. Due to the observational design and variable follow-up, outcomes were evaluated using a complete-case approach with phase-specific denominators.

Patient-reported symptom change (categorized as improvement, no change, and worsening) was described across predefined treatment phases (intermediate, end-of-treatment, and recall). Tolerability was assessed based on the proportion of patients reporting high-intensity symptom worsening (NRS ≥ 8) in the headache or generalized pain domains.

Comparisons between age groups (<45, 45–65, and >65 years) were performed at each predefined milestone using Pearson’s chi-square (χ^2^) test or Fisher’s exact test when appropriate. Proportions are presented with 95% confidence intervals (CI) calculated using Wilson’s method.

Exploratory analyses were conducted in patients who attended recall sessions to describe symptom evolution and tolerability during repeated exposure. These analyses were descriptive and were not subjected to formal hypothesis testing due to their non-protocolized and self-selected nature.

Missing data were not imputed. A two-sided *p*-value < 0.05 was considered statistically significant. All analyses were performed using Python version 3.9.

### 2.9. Use of Artificial Intelligence Tools

During the preparation of this manuscript, generative artificial intelligence tools (ChatGPT, OpenAI GPT-5 series) were used solely for language editing and text refinement. These tools were not used for data generation, data analysis, or interpretation of results. All AI-assisted content was critically reviewed, edited, and approved by the authors, who take full responsibility for the integrity and accuracy of the work.

## 3. Results

### 3.1. Study Flow and Cohort Characteristics

A total of 1446 clinical records were initially retrieved. After data cleaning to remove duplicate and incomplete entries, 1381 unique patients were included in the intention-to-treat (ITT) cohort (Table 1).

Of these, 821 patients reached the intermediate assessment and 554 completed the eight-session induction protocol. Additionally, 144 patients attended at least one patient-initiated recall session. Further recall visits were less frequent.

Reasons for treatment discontinuation were not systematically recorded, as data were derived from routine clinical practice.

The study population was predominantly female (89.7%), with a mean age of 54.16 ± 10.82 years. No significant differences were observed across centers in baseline characteristics or safety outcomes (*p* > 0.05).

Patient recruitment was uneven across participating centers, with two high-volume sites (Sevilla and Madrid) contributing approximately 49% and 43% of the cohort, respectively, while all other centres each accounted for less than 5% of cases (Supplementary Table S1).

Accordingly, comparisons across centres were interpreted descriptively. No significant differences were observed between high- and low-recruiting sites in baseline demographic characteristics (age and sex) or in safety outcomes, defined as the proportion of patients reporting transient high-intensity symptom worsening (NRS ≥ 8) (all *p* > 0.05).

### 3.2. Safety Outcomes Stratified by Age Group

Treatment-related transient symptom changes were evaluated using patient-reported assessments based on a 0–10 numerical rating scale (NRS), with a predefined threshold of ≥8 used to identify clinically relevant transient worsening. These outcomes were analyzed at two predefined treatment milestones—intermediate and end-of-treatment—and stratified by age group, as presented in Table 2.

Across both assessment phases, the proportion of patients reporting high-intensity symptom worsening (NRS ≥ 8) remained low in all age groups (Table 2). No statistically significant differences were observed between age strata at any milestone (all *p* > 0.05).

Reported events were clinically mild and transient, typically consisting of short-lived headache or generalized pain worsening. These symptoms were resolved spontaneously and did not require medical intervention, treatment modification, or discontinuation.

No serious adverse events were spontaneously reported or clinically identified during routine, non-systematic clinical follow-up across any assessment stage.

### 3.3. Patient-Reported Symptom Assessment and Tolerability Across Treatment Phases

Patient-reported symptom assessments across treatment phases are summarized in Table 3. These measures reflect perceived changes in symptoms based on a standardized 0–10 numerical rating scale (NRS) and should be interpreted as descriptive indicators rather than measures of clinical efficacy.

Across all treatment phases, most patients reported either improvement or no change, while the proportion reporting symptom worsening remained consistently low. Similar patterns were observed for pain-, headache-, and sleep-related domains.

The proportion of patients reporting high-intensity symptom worsening (NRS ≥ 8) was low at all timepoints and decreased across successive treatment phases. No serious adverse events were spontaneously reported or clinically identified during routine, non-systematic clinical follow-up.

Data from recall sessions were analyzed as exploratory observations. These sessions were patient-initiated and conducted after a variable follow-up period (median 202 days; range 48–363 days). No increase in symptom worsening or high-intensity responses was observed during repeated Li-rTMS exposure.

## 4. Discussion

The present multicentre observational cohort study provides large-scale real-world evidence on the safety and tolerability of low-intensity repetitive transcranial magnetic stimulation (Li-rTMS) in patients with fibromyalgia treated in routine outpatient settings. To our knowledge, this study includes one of the largest cohorts reported to date assessing Li-rTMS tolerability under real-world clinical conditions.

Across the evaluated population, Li-rTMS was associated with a low frequency of patient-reported transient high-intensity symptom worsening, with no serious adverse events spontaneously reported or clinically identified within the scope of routine outpatient follow-up. Reported symptom changes—headache, generalized pain, and sleep-related disturbances—were typically mild, self-limited, and resolved spontaneously within 24 h without requiring medical intervention. No seizures, syncopal events, hospitalizations, or other clinically significant complications were identified.

No statistically significant differences in high-intensity symptom worsening were observed across predefined age groups, including patients older than 65 years. However, given the low overall event rates and the absence of detailed data on comorbidities, frailty, and concomitant medication burden, these findings should be interpreted cautiously and considered exploratory in older populations.

Descriptive analyses across treatment phases did not indicate an increase in adverse symptom burden during repeated Li-rTMS exposure. Similarly, exploratory observations from patient-initiated recall sessions did not reveal late-emerging safety concerns. Nevertheless, these findings must be interpreted with caution, as recall assessments were voluntary, non-protocolized, and derived from a self-selected subgroup.

The tolerability profile observed in this study is consistent with previous reports on low-intensity neuromodulation approaches, which generally describe low rates of transient and mild patient-reported symptom worsening. Studies in fibromyalgia using Li-rTMS have typically involved small samples and controlled experimental settings, often reporting limited transient side effects, such as mild headache or transient discomfort. In parallel, evidence from conventional high-intensity rTMS indicates that procedural adverse events are generally infrequent and self-limited, although higher stimulation intensities may increase treatment-related discomfort and procedural complexity. Within this context, the present findings provide complementary real-world evidence from a substantially larger and more heterogeneous population. Direct quantitative comparisons across studies, however, remain limited by differences in design, stimulation parameters, and outcome definitions.

These observations are also consistent with recent clinical practice guidelines for rTMS, which emphasize standardized treatment protocols and the generally favourable safety profile of non-invasive brain stimulation techniques [18], although such guidelines typically rely on data from structured clinical trials rather than routine PMCF data.

Several important limitations must be considered when interpreting these results. First, the retrospective and observational design precludes causal inference and limits the ability to control for potential confounding variables, including concomitant pharmacological treatments and baseline clinical heterogeneity.

Second, a major limitation is the substantial attrition observed across treatment phases, with decreasing patient retention from baseline to intermediate and final assessments. Because reasons for discontinuation were not systematically recorded, it is not possible to exclude that patients experiencing unfavourable responses, transient symptom worsening, lack of perceived benefit, or practical barriers may have preferentially discontinued treatment. Consequently, the reported frequency of high intensity symptom worsening at later stages may be underestimated due to selective retention (survivorship bias). The present analyses were therefore conducted using phase-specific denominators (complete-case approach), which accurately reflect the characteristics of patients attending each timepoint but do not allow conclusions to be generalized to the entire initial cohort.

Third, safety outcomes were based on structured patient-reported assessments rather than a formal active adverse event surveillance system. As a result, detailed event-related attributes—such as precise onset timing, duration, formal severity grading, and standardized causality assessment—were not systematically captured, which represents a critical distinction from rigorous regulatory safety surveillance investigations. In addition, routine clinical data collection may be subject to underreporting of mild or transient events compared with controlled trial settings. Accordingly, the observed absence of serious adverse events should be interpreted cautiously as a favourable finding within available routine follow-up data rather than definitive evidence of the overall safety profile of Li-rTMS.

Fourth, patient recruitment was highly uneven across participating centres, with most cases originating from two high-volume sites. Although no relevant differences in baseline characteristics or safety outcomes were detected across centres, this imbalance limits the strength of between-centre comparisons and suggests that results are primarily driven by a subset of sites.

Fifth, the study lacked detailed baseline clinical variables such as disease duration, symptom severity scores, psychiatric comorbidities, and concomitant treatment profiles. While this reflects the pragmatic nature of real-world clinical registries, it limits the ability to perform adjusted analyses and to fully contextualize the representativeness of the cohort.

Finally, the predominantly female composition of the study population reflects the epidemiology of fibromyalgia but may limit generalizability to male patients.

Despite these limitations, the study provides valuable real-world evidence supporting the feasibility and tolerability of Li-rTMS in routine clinical practice. The low frequency of transient symptom exacerbations, the absence of serious adverse events in available follow-up data, and the consistency of findings across treatment phases suggest that Li-rTMS represents a manageable and well-tolerated non-invasive neuromodulation approach within the subset of patients who continue treatment.

Future prospective studies with active standardized adverse event surveillance frameworks, systematic follow-up of treatment discontinuation, and controlled designs are required to more precisely quantify safety, identify predictors of tolerability, and establish the clinical role of Li-rTMS in fibromyalgia and related chronic pain conditions.

From a technical and clinical perspective, Li-rTMS differs substantially from conventional high-intensity rTMS, as it operates at markedly lower magnetic field strengths and does not require suprathreshold stimulation or motor threshold calibration. These characteristics may contribute to improved tolerability and simplified clinical implementation. However, comparative conclusions regarding safety or efficacy between Li-rTMS and conventional rTMS cannot be established based on the present observational data and should be addressed in dedicated controlled studies.

## 5. Conclusions

In this large multicenter real-world cohort of patients with fibromyalgia, Li-rTMS demonstrated a favorable patient reported tolerability profile specifically among the subset of patients with available follow-up data in a real-world, routine outpatient practice setting. No increase in perceived symptom burden was identified during repeated exposure observations, including exploratory patient-initiated recall sessions. However, due to the high attrition rates clinical conclusions regarding universal tolerability must be framed cautiously, and further prospective studies with standardized drop-out tracking are warranted. Importantly, these findings reflect patient-reported tolerability outcomes collected during routine clinical practice rather than a formally adjudicated adverse event surveillance framework.

In conclusion, low-intensity repetitive transcranial magnetic stimulation (Li-rTMS) demonstrated a highly favorable documented tolerability profile among the substantial subgroup of fibromyalgia patients with available follow-up milestones within routine clinical care. While the lack of a control group, the absence of an active safety surveillance protocol and the presence of treatment attrition limit the generalizability of these findings to the entire initial intention-to-treat cohort, the real-world data suggest that Li-rTMS represents a manageable, non-invasive option for patients who maintain adherence to the multi-session protocol in routine clinical practice.

## Figures and Tables

**Table 1:** Study flow, treatment retention, and follow-up availability across study phases.

Study Phase	N (Unique Patients)	Percentage Relative to Baseline Cohort (N = 1381)	Observation
Pre-treatment assessment ^1^	1381	100%	Baseline cohort
Intermediate assessment	821	59.44%	Treatment Retention
End-of-treatment assessment	554	40.11%	Treatment Retention
Recall Session 1 (R1)	144	10.42%	Follow-up Availability ^2^
Recall Session 2 (R2)	32	2.31%	Follow-up Availability
Recall Session 3+ (R3+)	11	0.79%	Follow-up Availability (three or more sessions)

^1^ A total of 31 cases (65 entries) were excluded from the initial 1446 records due to duplicate entries to ensure data integrity. ^2^ Recall sessions were patient-initiated following a minimum 30-day interval post-treatment. Percentages for R1-R3+ are calculated over the total baseline cohort to show overall treatment engagement.

**Table 2:** Treatment-related transient symptom changes stratified by milestone phase and age group.

High-Intensity Symptom Worsening (NRS ≥ 8) ^1^	<45 Years (Inter: n = 235/ Final: n = 144)	45–65 Years (Inter: n = 503/ Final: n = 349)	>65 Years (Inter: n = 83/Final: n = 61)	*p*-Value (Inter) ^2^	*p*-Value (Final) ^3^
Headache				0.24	0.58
Intermediate Phase	12/235 (5.1%) [2.9–8.7]	19/503 (3.8%) [2.4–5.9]	3/83 (3.2%) [1.0–9.5]		
End-of-Treatment Phase	3/144 (2.3%) [0.8–6.3]	10/349 (3.0%) [1.7–5.4]	2/61 (2.4%) [0.5–9.9]		
Generalized Pain				0.47	0.62
Intermediate Phase	13/235 (5.6%) [3.3–9.3]	32/503 (6.4%) [4.6–8.9]	5/83 (5.9%) [2.5–13.2]		
End-of-Treatment Phase	7/144 (4.7%) [2.3–9.5]	17/349 (5.0%) [3.2–7.8]	2/61 (3.5%) [1.0–11.5]		
Sleep Disturbance				0.68	0.49
Intermediate Phase	5/235 (2.2%) [1.0–5.0]	10/503 (1.9%) [1.0–3.5]	2/83 (2.7%) [0.8–8.8]		
End-of-Treatment Phase	2/144 (1.2%) [0.3–4.6]	5/349 (1.4%) [0.6–3.3]	1/61 (1.2%) [0.2–8.0]		
Serious Adverse Events (SAEs) Spontaneously Reported or Clinically Identified During Routine Follow-up					
Intermediate Phase	0/235 (0%)	0/503 (0%)	0/83 (0%)		
End-of-Treatment Phase	0/144 (0%)	0/349 (0%)	0/61 (0%)		

^1^ Significant transient symptom worsening defined as NRS ≥ 8. The identification of serious adverse events (SAEs) was based on spontaneous patient reports and routine clinical follow-up rather than on a formal active safety surveillance system. ^2^ *p*-value derived from the Pearson chi-square test (χ^2^), analyzing the intergroup distribution by age ranges in the intermediate phase. ^3^ *p*-value derived from the Pearson chi-square test (χ^2^), analyzing the intergroup distribution by age ranges at the end of treatment. Percentages are calculated using phase-specific denominators. Notes: Data are presented as n/N (%), with 95% confidence intervals (CI) calculated using Wilson’s score method in brackets. Percentages and confidence intervals correspond to the originally computed values. Counts (n) have been derived from reported percentages and denominators and may not exactly reproduce CI limits due to rounding. Percentages are based on phase-specific denominators (complete-case analysis), corresponding to the number of patients with available data at each assessment phase.

**Table 3:** Patient-reported perceived symptom change and tolerability across treatment phases.

Clinical Parameter	Intermediate (n = 821)	End of Treatment (n = 554)	Recall Session ^f^ (R1, n = 144)
Pain-Related Symptom Assessment			
Improvement (NRS 0–4) ^a^	432/821 (52.70%)	421/554 (76.10%)	103/144 (71.40%)
Stable/No Change (NRS 5) ^b^	355/821 (43.20%)	120/554 (21.60%)	37/144 (25.50%)
Symptom Worsening (NRS 6–10) ^c^	34/821 (4.10%)	13/554 (2.30%)	4/144 (3.10%)
Headache-Related Symptom Assessment			
Improvement (NRS 0–4) ^a^	485/821 (58.20%)	40/554 (72.40%)	99/144 (68.90%)
Sleep-Related Symptom Assessment			
Improvement (NRS 0–4) ^a^	409/821 (49.80%)	357/554 (64.50%)	88/144 (61.20%)
Tolerability Indicator			
Transient Symptom Exacerbations (NRS ≥ 8) ^d^	51/821 (6.24%)	12/554 (2.14%)	3/144 (2.08%)
	[4.79–8.10]	[1.23–3.71]	[0.71–5.95]
Stratified by Age Group			
<45 years	16/235 (6.77%)	4/144 (2.48%)	1/52 (1.92%)
	[4.48–10.12]	[1.06–5.67%]	[0.34–10.15]
45–65 years	29/503 (5.78%)	7/349 (1.89%)	1/69 (1.45%)
	[3.91–8.46%]	[0.81–4.36]	[0.26–7.77]
>65 years	5/83 (6.40%)	1/61 (2.13%)	1/23 (4.35%)
	[3.28–12.11]	[0.58–7.44]	[0.77–21.01]
Age-group Comparison (*p*-value) ^e^	*p* = 0.868	*p* = 0.897	*p* = 0.708
Serious Adverse Events (SAEs) Spontaneously Reported or Clinically Identified During Routine Follow-up	0%	0%	0%

^a^ Improvement corresponds to patient-reported scores of 0–4 on the treatment assessment scale (0 = maximum improvement, 10 = maximum worsening). ^b^ Stable/No change corresponds to a neutral score of 5 on the assessment scale. ^c^ Symptom worsening corresponds to scores of 6–10 on the assessment scale. ^d^ Transient symptom exacerbations were defined as acute high-intensity responses (NRS ≥ 8) in headache- or pain-related assessments during the corresponding treatment phase. Serious adverse events reflect the absence of major treatment-related complications spontaneously reported or clinically identified during routine, non-systematic outpatient follow-up. ^e^ *p*-values were computed using Pearson’s chi-square (Χ^2^) test to compare the distribution of transient exacerbations across the three age strata (<45, 45–65, and >65 years) within each specific treatment phase. ^f^ Recall sessions were patient-initiated and analysed descriptively as exploratory observations under routine clinical practice conditions. Notes: The assessment scale reflects perceived change relative to pre-treatment symptom status rather than absolute symptom intensity. Percentages are calculated using phase-specific denominators (complete-case analysis), corresponding to the number of patients with available data at each treatment phase (Intermediate: n = 821; End-of-treatment: n = 554; Recall: n = 144), rather than the full intention-to-treat (ITT) cohort.

## Data Availability

The data presented in this study are available on request from the corresponding author.

## Supplementary Materials

The following supporting information can be downloaded at: https://www.mdpi.com/article/10.3390/jcm15124452/s1, Table S1: Distribution of patients across participating centers (Pre-treatment assessment, N = 1381).

## Abbreviations

The following abbreviations are used in this manuscript:

ACR	American College of Rheumatology
AE	Adverse Event
AEMPS	Spanish Agency of Medicines and Medical Devices
CE	Conformité Européenne
FM	Fibromyalgia
GDPR	General Data Protection Regulation
ITT	Intention-to-Treat
Li-rTMS	Low-Intensity Repetitive Transcranial Magnetic Stimulation
MDR	Medical Device Regulation
NRS	Numerical Rating Scale
PMCF	Post-Market Clinical Follow-Up
SAE	Serious Adverse Event
SD	Standard Deviation
STROBE	Strengthening the Reporting of Observational Studies in Epidemiology
TAE	Transient Adverse Event

## References

[1] Clauw D.J. (2014). Fibromyalgia: A Clinical Review. JAMA.

[2] Wolfe F., Clauw D.J., Fitzcharles M.A., Goldenberg D.L., Häuser W., Katz R.L., Mease P., Russell A.S., Russell I.J., Walitt B. (2010). The American College of Rheumatology Preliminary Diagnostic Criteria for Fibromyalgia and Measurement of Symptom Severity. Arthritis Care Res..

[3] Häuser W., Fitzcharles M.A. (2018). Fibromyalgia: Ongoing Challenges and Recent Advances. Am. J. Med..

[4] Walitt B., Ceko M., Gracely J.L., Gracely R.H. (2016). Neuroimaging of Central Sensitivity Syndromes: Key Insights from the Scientific Literature. Curr. Rheumatol. Rev..

[5] Boomershine C.S. (2015). Fibromyalgia: The Prototypical Central Sensitivity Syndrome. Curr. Rheumatol. Rev..

[6] Schmidt-Wilcke T., Clauw D.J. (2011). Fibromyalgia: From Pathophysiology to Therapy. Nat. Rev. Rheumatol..

[7] Häuser W., Sarzi-Puttini P., Fitzcharles M.A. (2019). Fibromyalgia Syndrome: Under-, Over- and Misdiagnosis. Clin. Exp. Rheumatol..

[8] Lefaucheur J.P., Aleman A., Baeken C., Benninger D.H., Brunelin J., Di Lazzaro V., Filipović S.R., Grefkes C., Hasan A., Hummel F.C. (2020). Evidence-Based Guidelines on the Therapeutic Use of Repetitive Transcranial Magnetic Stimulation (rTMS): An Update (2014–2018). Clin. Neurophysiol..

[9] Zhang J.-H., Liang J., Yang Z.-W. (2023). Non-invasive brain stimulation for fibromyalgia: Current trends and future perspectives. Front. Neurosci..

[10] Velickovic Z., Radunovic G. (2024). Repetitive transcranial magnetic stimulation in fibromyalgia: Exploring the necessity of neuronavigation for targeting new brain regions. J. Pers. Med..

[11] Passard A., Attal N., Benadhira R., Brasseur L., Saba G., Sichere P., Perrot S., Januel D., Bouhassira D. (2007). Effects of Unilateral Repetitive Transcranial Magnetic Stimulation of the Motor Cortex on Chronic Widespread Pain in Fibromyalgia. Brain.

[12] Makowiecki K., Garrett A., Harvey A.R., Rodger J. (2018). Low-Intensity Repetitive Transcranial Magnetic Stimulation Requires Concurrent Visual System Activity to Modulate Visual Evoked Potentials in Adult Mice. Sci. Rep..

[13] Moretti J., Rodger J. (2022). A Little Goes a Long Way: Neurobiological Effects of Low Intensity rTMS and Implications for Mechanisms of rTMS. Curr. Res. Neurobiol..

[14] Maestú C., Blanco M., Nevado A., Romero J., Rodríguez-Rubio P., Galindo J., Lorite J.B., Morenas F.d.L., Fernández-Argüelles P. (2013). Reduction of Pain Thresholds in Fibromyalgia after Very Low-Intensity Magnetic Stimulation: A Double-Blinded, Randomized Placebo-Controlled Clinical Trial. Pain Res. Manag..

[15] Gomez-Arguelles J.M., Travesi B., Maestu C. (2022). Modification in Pittsburgh Sleep Quality Index after Low Intensity Transcranial Magnetic Stimulation in Patients with Fibromyalgia. Clin. Exp. Rheumatol..

[16] Pareja J.L., Cáceres O., Zambrano P., Martín F., Berral F.J., Blanco M. (2022). Treatment with Low-Intensity Transcranial Magnetic Stimulation in Women with Fibromyalgia Improves Diagnostic Variables up to 6 Months after Treatment Completion. Clin. Exp. Rheumatol..

[17] Felix-Gonzalez N. (2026). nazariofelix-CTB/fibromyalgia-li-rtms-pmcf-dataset: PMCF Fibromyalgia Li-rTMS Dataset and Questionnaire.

[18] Hussain S., Chamoli S., Fitzgerald P., Gandhi A., Gill S., Sarma S., Loo C. (2024). Royal Australian and New Zealand College of Psychiatrists professional practice guidelines for the administration of repetitive transcranial magnetic stimulation. Aust. N. Z. J. Psychiatry.

