# Supplementary material for: Real-World Safety and Tolerability of Low-Intensity Repetitive Transcranial Magnetic Stimulation in Fibromyalgia: A Multicenter Observational Cohort Study
Source: J Clin Med. 2026 Jun 9;15(12):4452. doi: 10.3390/jcm15124452 (PMC13301379; doi:10.3390/jcm15124452)
Supplement: Supplementary file 1 [file jcm-15-04452-s001.zip › jcm-4336928-supplementary.pdf]

Table S1. Distribution of patients across participating centers (Pre-treatment assessment, N = 1,381)

Center (anonymized by location)	N	% of Data
Center 1(Sevilla)	677	49.0
Center 2 (Madrid)	596	43.2
Center 3(Barcelona)	48	3.5
Center 4(Madrid)	32	2.3
Centers 5-9 (combined)	28	2.0
**TOTAL**	1,381	100.0
